# Aberrant Expression of Posterior *HOX* Genes in Well Differentiated Histotypes of Thyroid Cancers

**DOI:** 10.3390/ijms141121727

**Published:** 2013-11-01

**Authors:** Monica Cantile, Giosuè Scognamiglio, Lucia La Sala, Elvira La Mantia, Veronica Scaramuzza, Elena Valentino, Fabiana Tatangelo, Simona Losito, Luciano Pezzullo, Maria Grazia Chiofalo, Franco Fulciniti, Renato Franco, Gerardo Botti

**Affiliations:** 1 Pathology Unit, National Cancer Institute “G. Pascale” Foundation, via Mariano Semmola 80131, Napoli, Italy; E-Mails: monicantile@libero.it (M.C.); giosue.s@virgilio.it (G.S.); lucia.ls@hotmail.it (L.L.S.); doctorelvy.lm@gmail.com (E.L.M.); cantilemo@yahoo.it (V.S.); renfr@yahoo.com (E.V.); f.tatangelo@istitutotumori.na.it (F.T.); s.losito@istitutotumori.na.it (S.L.); f.fulciniti@istitutotumori.na.it (F.F.); g.botti@istitutotumori.na.it (G.B.); 2 Thyroid and Parathyroid Surgery Unit, National Cancer Institute “G. Pascale” Foundation, via Mariano Semmola 80131, Napoli, Italy; E-Mails: l.pezzullo@istitutotumori.na.it (L.P.); m.chiofalo@istitutotumori.na.it (M.G.C.)

**Keywords:** *HOX* 13 paralogous genes, thyroid histotypes, thyroid tumors progression

## Abstract

Molecular etiology of thyroid cancers has been widely studied, and several molecular alterations have been identified mainly associated with follicular and papillary histotypes. However, the molecular bases of the complex pathogenesis of thyroid carcinomas remain poorly understood. *HOX* genes regulate normal embryonic development, cell differentiation and other critical processes in eukaryotic cell life. Several studies have shown that *HOX* genes play a role in neoplastic transformation of several human tissues. In particular, the genes belonging to *HOX* paralogous group 13 seem to hold a relevant role in both tumor development and progression. We have identified a significant prognostic role of *HOX D13* in pancreatic cancer and we have recently showed the strong and progressive over-expression of *HOX C13* in melanoma metastases and deregulation of *HOX B13* expression in bladder cancers. In this study we have investigated, by immunohistochemisty and quantitative Real Time PCR, the *HOX* paralogous group 13 genes/proteins expression in thyroid cancer evolution and progression, also evaluating its ability to discriminate between main histotypes. Our results showed an aberrant expression, both at gene and protein level, of all members belonging to paralogous group 13 (*HOX A13*, *HOX B13*, *HOX C13* and *HOX D13*) in adenoma, papillary and follicular thyroid cancers samples. The data suggest a potential role of *HOX* paralogous group 13 genes in pathogenesis and differential diagnosis of thyroid cancers.

## 1. Introduction

Thyroid tumors are the most common tumors of the endocrine system (1% of all cancers). Approximately 94% of thyroid cancers are well-differentiated forms (papillary and follicular), 5% are represented by medullary carcinoma and the remaining 1% anaplastic carcinomas [1]. In recent years the progressive knowledge of the molecular mechanisms involved in thyroid transformation have allowed the association of alterations of specific markers to several thyroid tumor histotypes [2] permitting the formulation of a rather accurate model of tumorigenesis, although not yet fully defined [3]. Rearrangements of *RET*, for example, are specific for papillary carcinoma [4], and mutations of *B-RAF* are also associated to a more aggressive course [5]. However, the prognosis of thyroid cancer remains unpredictable, thus the identification of new biological markers are needed in addition to already known molecules, to correctly stratify patients at risk of recurrence and progression.

*HOX* genes regulate normal embryonic development, cell differentiation and other critical processes in eukaryotic cell life. Several studies have demonstrated that *HOX* genes play a role in neoplastic transformation in several human tissues [6–10]. In particular, the genes belonging to HOX paralogous group 13 seem to carry out a relevant role in both tumor development and progression.

We have identified a significant prognostic role of *HOX D13* in pancreatic cancer [11] and the *HOX A13* gene deregulation in liver carcinogenesis [12]. The amplification of 12q13–15 locus, containing *HOX C13* gene, was associated with a large number of cancers such as sarcomas, glioblastomas, lung, bladder and transitional carcinomas and melanomas [13–20]. Moreover, we have recently showed the strong and progressive over-expression of *HOX C13* in melanoma metastases when compared to nevi and primary melanomas, suggesting its role in a metastatic melanoma switch [21]. We have also showed the aberrant expression of *HOX B13* in bladder tumorigenesis and progression [22].

In this study we have analyzed paralogous 13 *HOX* genes expression, by immunohistochemistry and real time PCR, in non-neoplastic thyroid samples, in a case series of thyroid cancer histotypes and in the corresponding lymph nodal metastases, highlighting a potential role of these genes in thyroid cancer evolution.

## 2. Results

### 2.1. Clinic Pathological Characteristics of Thyroid Cancers and Patients

In our series of tissue samples, 50 specimens of thyroid cancers and 12 normal thyroid tissues as controls are included. Tumor samples comprised two main histotypes: classical papillary carcinoma (CPC) (50%), follicular variant papillary carcinoma (FPC) (32%), and follicular carcinoma (FC) (13%).

Female patients were 38 out of 50 (76%), while male were 12 (24%). The age range was 13–74 years, with an average of 43 years. Regarding the staging, 58% was stage I, 4% stage II, 26% stage III, and 13% stage IV. The details of clinic-pathological data are shown in Table 1.

### 2.2. Paralogous 13 *HOX* Genes Expression in Thyroid Cancer Histotypes

**Figure 1:**
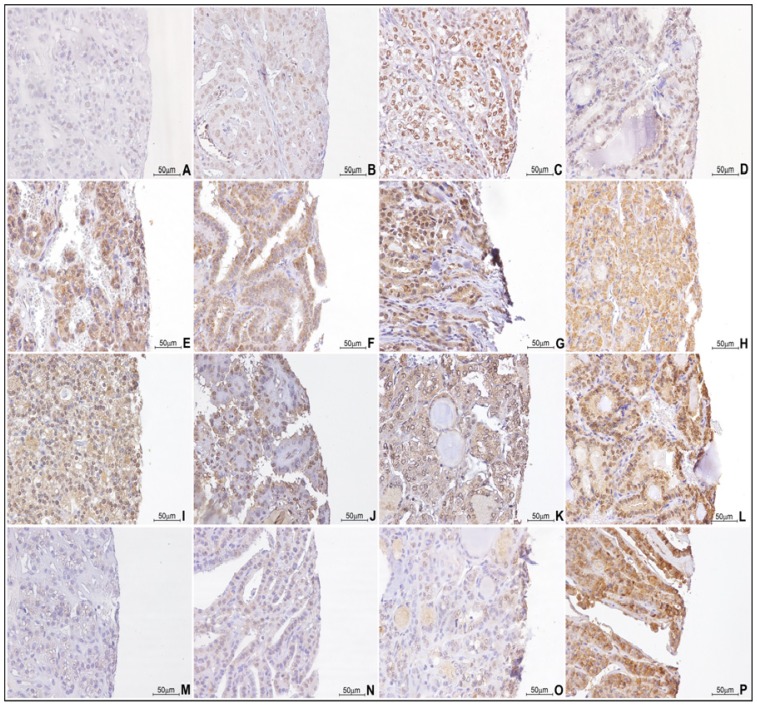
Paralogous group 13 *HOX* genes IHC expression in thyroid samples: (**A**) *HOX A13* expression in normal tissue (40×); (**B**) *HOX A13* expression in adenoma (40×); (**C**) *HOX A13* expression in CPC histotype (40×); (**D**) *HOX A13* expression in FC histotype (40×); (**E**) *HOX B13* expression in normal tissue (40×); (**F**) *HOX B13* expression in adenoma (40×); (**G**) *HOX B13* expression in CPC histotype (40×); (**H**) *HOX B13* expression in FC histotype (40×); (**I**) *HOX C13* expression in normal tissue (40×); (**J**) *HOX C13* expression in adenoma (40×); (**K**) *HOX C13* expression in CPC histotype (40×); (**L**) *HOX C13* expression in FC histotype (40×); (**M**) *HOX D13* expression in normal tissue (40×); (**N**) *HOX D13* expression in adenoma (40×); (**O**) *HOX D13* expression in CPC histotype (40×); (**P**) *HOX D13* expression in FC histotype (40×).

*HOX A13*, *HOX B13*, *HOX C13* and *HOX D13* expressions were detected by immunohistochemistry on t-MTA. We considered both nuclear and cytoplasmic positivity for statistical associations. In general, tumor samples stained more consistently for paralogous 13 *HOX* genes than their normal counterpart (normal tissues and adenomas) (Figure 1).

**Figure 2:**
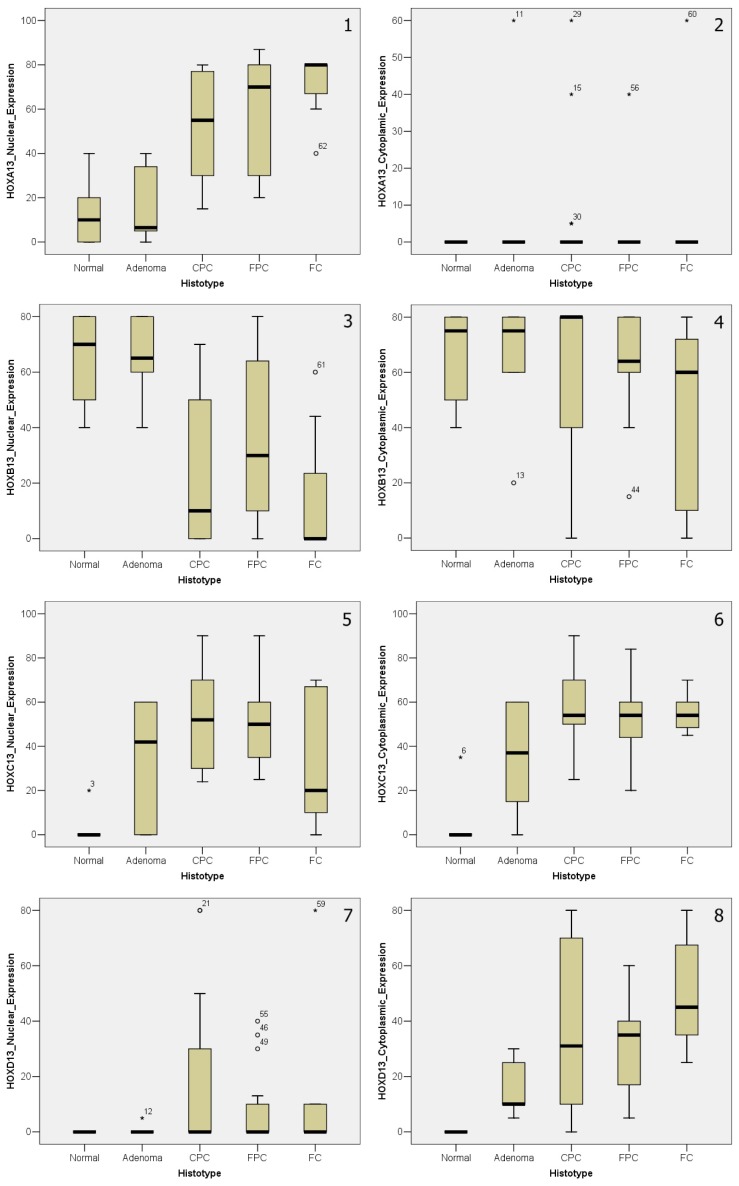
Plot graphic representation of paralogous group 13 *HOX* genes IHC expression in thyroid samples: (**1**,**2**) Nuclear (on the **left**) and cytoplasmic (on the **right**) *HOX A13* expression; (**3**,**4**) Nuclear (on the **left**) and cytoplasmic (on the **right**) *HOX B13* expression; (**5**,**6**) Nuclear (on the **left**) and cytoplasmic (on the **right**) *HOX C13* expression; (**7**,**8**) Nuclear (on the **left**) and cytoplasmic (on the **right**) *HOX D13* expression (^*^ and ° indicate values off the graph).

All IHC *HOX* expression data were statistically analyzed and all statistical elaborations are schematized in (Table S1) and represented in graphic form in Figure 2.

Aberrant *HOX A13* nuclear expression appeared strongly associated (*p <* 0.001) to different histotypes of thyroid cancer. Its expression gradually increased from adenoma, to CPC, to FPC, until FC. The opposite trend characterized *HOX B13*, a significant decreasing (*p* = 0.0013) of nuclear expression in the transition from non-neoplastic tissues to different tumor histologic types. Regarding *HOX C13*, both nuclear (*p =* 0.0021) and cytoplasmic (*p =* 0.0013) expression were significant, with a trend of increasing from the normal thyroid to the adenoma and to cancer histologic types, except for a reverse trend of the only nuclear expression in FC histotype. Finally, a strong statistical significance was shown for cytoplasmic expression (*p <* 0.001) of *HOX D13*, that gradually increased from normal tissues, to adenoma, to CPC, to FPC, until FC.

The comparison between the nuclear and cytoplasmic expression between 4 markers, associated in pairs, showed a predominant tendency to cytoplasmic localization as shown in Table 2. The detail of the trends of expression is shown in Table S2.

Paralogous 13 *HOX* genes expression was also detected by real time PCR on 10 thyroid samples, 2 adenoma, 4 CPC, 2 FPC, and 2 FC histotypes.

**Figure 3:**
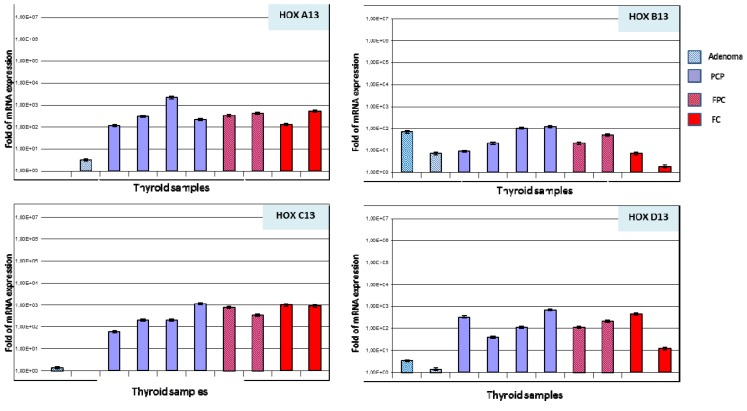
Real Time PCR expression of *HOX A13*, *HOX B13*, *HOX C13* and *HOX D13* genes in thyroid tissue samples. All reactions were performed in triplicate and data are expressed as mean of relative amount of mRNAs levels.

A trend of increasing expression was observed between non-neoplastic tissues and various thyroid tumor histotypes, as showed by immunohistochemical analysis. In particular, normal tissue and adenoma showed a low or absent expression (between 0–10 fold expression) particularly for *HOX A13*, *HOX C13* and *HOX D13*, whereas, in all tumor samples an increased expression was detected (between 100–1000 fold expression). Moreover, for *HOX B13* no significant differences were present between adenoma and CPC histotype with a moderate expression (between 10–100 fold expression), while FC histotype showed a low expression (between 0–10 fold expression) (Figure 3).

### 2.3. Paralogous 13 *HOX* Genes Expression in Papillary and Follicular Thyroid Cancer Progression

**Figure 4:**
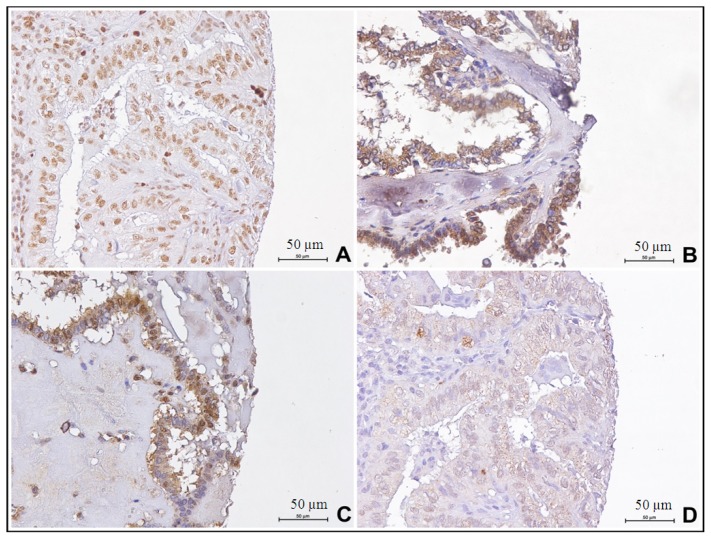
Paralogous group 13 *HOX* genes IHC expression in metastatic thyroid samples: (**A**) *HOX A13* expression (40×); (**B**) *HOX B13* expression (40×); (**C**) *HOX C13* expression (40×); (**D**) *HOX D13* expression (40×).

Nuclear and cytoplasmic *HOX* expression was detected by immunohistochemistry and statistically analysed on thyroid samples for which we had primary tumors and corresponding lymph node metastases (Figure 4).

All IHC results statistically elaborated, are shown in Table S3, but no significant association has been found.

## 3. Discussion

Various evidence associating the altered expression of many *HOX* genes to cancerogenesis have previously been shown [8].

In this study we have focused attention on *HOX* genes of paralogous group 13, with activity correlated to cell proliferation, and for which deregulation is often associated with tumor evolution [11,12,21–25].

Genes posteriorly located in the *HOX* network (paralogous 13 *HOX* genes) are responsible of the correct formation of limbs and urogenital structures during the normal development. When mutated, they can generate life-compatible phenotypes, such as synpolydactyly and hand-foot-genital syndrome [26,27].

Among paralogous 13 *HOX* genes, *HOX* A13 expression has been associated to enhance tumor growth *in vitro*, and *in vivo* to tumor-node-metastasis (TNM) stage and disease-free survival time of patients with esophageal squamous cell carcinoma (ESCC) [28]. Moreover, its overexpression has been detected in primary hepatocarcinoma and *versus* non-tumorous livers [12]. Recently, an up-regulation of *HOXA13* expression has been associated with highly aggressive forms of gastric cancer, highlighting its prognostic role also in this type of cancer [29].

*HOX B13* has been associated with tumor evolution and progression of several hormone-dependent tumors, as prostate, ovary and breast cancers [30–32]. Recently, a rare, recurrent mutation (G84E) in *HOX B13* was reported to be associated with prostate cancer risk [33]; its aberrant expression was also shown in melanoma, cervical and colon cancers [34–36]. Finally, we recently showed that *HOX B13* aberrant expression might better stratify patients with non-muscle invasive bladder cancers (NMIBCs) at risk of recurrence [22]. Posterior locus C *HOX* genes have been associated to bladder and renal cancer [37,38] and the strong and progressive over-expression of *HOX C13* in melanoma metastases has suggested its potential role in metastatic melanoma switch [21]. Finally, *HOX D13* deregulation has been reported in several human cancers, and its aberrant expression was strongly associated to pancreatic cancer development and progression [11].

However, there are few suggestions about the role of *HOX* genes in the development and progression of thyroid tumors based on cellular models [39]. Although thyroid papillary cancer generally manifests an indolent biologic behaviour, it exhibits, as FC histotype, a high degree of genomic instability [40], particularly chromosome rearrangements, [41] gene mutations [42] and de-regulation of many subsets of molecules, especially transcription factors [43–45]. Several studies have demonstrated that all these genetic alterations represent molecular prognosticators for these types of tumor.

We have analyzed paralogous 13 *HOX* genes expression in adenoma, CPC and FC thyroid histotypes. Our data showed, both at protein and gene level, an aberrant expression of genes of paralogous 13 in thyroid cancers. In fact, the four markers appear differently expressed in adenoma *versus* CPC (mainly) and FC histotypes, and between the two main histological tumor types.

Even if the two expression profiles are often not comparable, our immunohistochemistry data were confirmed by qRT-PCR, and this has allowed us to suggest that alterations in *HOX* expression are found at both the gene and protein level.

Moreover, we selected a case series of thyroid cancers with corresponding lymph nodal metastases, to analyze paralogous 13 *HOX* genes expression and to verify if the aberrant expression of these genes could also be associated with tumor progression. However, all paralogous 13 *HOX* genes were not significantly associated to lymph node metastases during thyroid tumor progression.

In our study we have separately considered nuclear and cytoplasmic HOX protein expressions. In fact, in most of the studies performed through immunohistochemistry, HOX proteins expression in the cytoplasm was often reported [8], nuclear expression was considered indicative, being transcriptional regulators [13].

The overall analysis of our data has indicated a trend of the four markers predominantly localized in the cytoplasm. The ability of these genes to present a cytoplasmic localization was already described in several tissues, during development [46], and in cancer [23]. In some types of cancer this different sub cellular localization has represented an unfavorable prognostic factor in tumor progression [23,25]. The different expressions, nuclear and cytoplasmic, of the 4 markers, were crossed with each other, showing a specific trend for the adenoma and for the two tumor histotypes.

In particular, the nuclear positivity of *HOX A13* and *HOX B13* showed an opposite trend in the transition from adenoma to cancer.

The differential diagnosis between adenomas and thyroid tumors often represents an important diagnostic problem. The use of *HOX A13* and *HOX B13* could suggest a clinical application, as a useful diagnostic tool, especially for cytological diagnosis.

## 4. Methods

### 4.1. Thyroid Cancer Patients

Fifty patients admitted to the National Cancer Institute “Giovanni Pascale” of Naples, between 2008 and 2011, were recruited in this study. All patients provided written informed consent for the use of samples according to the institutional regulations, and the study was approved by the ethics committee of the National Cancer Institute “G. Pascale”.

Normal and adenoma thyroid tissues and cells were used as non-neoplastic controls. All tumour cases have been reviewed according to WHO/ISUP 2007 classification criteria, using standard tissue sections. Medical records have been reviewed for clinical information, including histologic parameters assessed on standard H & E-stained slides.

### 4.2. TMAs Building

A thyroid Multi-Tumor Array (t-MTA) was constructed using 50 tumor tissue samples (7 FC and 43 CPC histotypes) and 12 non-neoplastic tissues samples (6 normal and 6 adenoma). The corresponding lymph node metastases for 14 samples from this series were also available. The most representative areas of surgical samples blocks were used for TMA construction. All tumors and controls were reviewed by two experienced pathologists (RF and SL). Discrepancies for the same case were resolved in a joint analysis. Tissue cylinders with a diameter of 1 mm were punched from morphologically representative tissue areas of each “donor” tissue block and brought into one recipient paraffin block (3 × 2.5 cm) using a semi-automated tissue arrayer (Galileo TMA, Integrated Systems Engineering, Milano, Italy).

### 4.3. Immunohistochemistry Analysis

Immunohistochemical staining was carried out on slides from formalin-fixed, paraffin embedded tissues, in order to evaluate the expression of *HOX A13*, *HOX B13*, *HOX C13* and *HOX D13*. Paraffin slides was then deparaffinized in xylene and rehydrated through graded alcohols. Antigen retrieval was performed with slides heated in 0.01 M citrate buffer (pH 6.0) in a bath for 20 min at 97 °C. After antigen retrieval, the slides were allowed to cool. The slides were rinsed with TBS and the endogenous peroxidase has inactivated with 3% hydrogen peroxide. After protein block (BSA 5% in PBS 1×), the slides were incubated with primary antibody to human *HOX A13* (dilution 1:200, cod. Ab106503, Abcam, Cambridge, UK), *HOX B13* (dilution 1:300, cod. ab28575, Abcam, Cambridge, UK), *HOX C13* (dilution 1:1200, cod.ab55251, Abcam, Cambridge, UK), *HOX D13* (dilution 1:100, cod. Ab19866, Abcam, Cambridge, UK) overnight. Sections were incubated with mouse anti-rabbit or goat anti-mouse secondary IgG biotinylated secondary antibody for 30 min. Immunoreactivity was visualized by means of avidin-biotin-peroxidase complex kit reagents (Novocastra, Newcastle, UK) as the chromogenic substrate. Finally, sections were weakly counterstained with haematoxylin and mounted.

### 4.4. Evaluation of Immunostaining

Antigen expression was independently evaluated by two experienced pathologists (RF/SL) using light microscopy. For paralogous 13 *HOX* genes, nuclear and cytoplasmic localization were considered. All values of immunostaining were expressed only in percentage terms of positive cells. The percentage of positive cancer cells was evaluated in each sample by counting the number of positive cells over the total cancer cells in 10 non-overlapping fields using ×400 magnification.

### 4.5. RNA Extraction and Analysis

Total RNA was isolated from 10 thyroid samples: 2 adenoma, 4 primary CPC, 2 FPC, and 2 FC histotypes. FFPE samples were collected from the National Cancer Institute “Fondazione G. Pascale” Institutional Bio-Bank. High pure FFPE RNA Micro Kit (Roche Molecular Biochemicals, Mannheim, Germany) was used for FFPE specimens following the manufacturer’s instructions. A total of 1 μg RNA was subjected to cDNA synthesis for 1 h at 37 °C using the Ready To Go You-Primer First-Strand Beads kit (Amersham Biosciences Europe Gmbh, Freiburg, Germany, cod.27-9264-01) in a reaction mixture containing 0.5 μg random hexamers (GeneAmp RNA PCR Random Hexamers Set N808-0127 Applied Biosystems, Foster City, CA, USA).

### 4.6. Quantitative Real-Time PCR

Quantitative RT-PCR was performed in a LightCycler system (Roche Molecular Biochemicals, Mannheim, Germany) using TaqMan^®^ analysis. In this system, all reactions have been run in glass capillaries in a volume of 20 μL with 4 μL of The LightCyclerTaqMan Master Mix (cod. 04735536001, Roche Molecular Biochemicals, Rotkreuz, Switzerland), 2 μL of cDNA and 1 μL of specific TaqMan Gene Expression Assays for human *HOX A13*, *HOX B13*, *HOX C13*, *HOX D13* (RealTime Designer Assay cod.05583055001, Roche Molecular Biochemicals, Rotkreuz, Switzerland) according to the manufacturer’s directions. All reactions were performed in triplicate. The thermal cycling conditions included a step of 20 s at 95 °C followed by a 40 cycles of 95 °C for 1 s and 60°C for 20 s. The comparative *C*_t_ method was employed to determine the human *HOX* genes variation, using TaqMan Endogenous Controls Human ACTB (β-actin) Endogenous Control (Real Time Designer Assay cod.05532957001, Roche Molecular Biochemicals, Rotkreuz, Switzerland) as reference gene. Final amounts of target were determined as follows: target amount = 2 − *C*_t_ where *C*_t_ = [*C*_t_ (*HOX* genes) − *C*_t_ (ACTB)]_sample_ − [*C*_t_ (*HOX* genes) − *C*_t_ (ACTB)]_calibrator_. Data were expressed as mean ± standard deviation (SD, *n =* 3).

### 4.7. Statistical Analysis

Only a percentage of immunoreactive cells were considered for the evaluation of paralogous *HOX* 13 IHC expression on t-MTA, encopassing different hystotypes.

The Kruskal-Wallis test was applied to identify differences in median expression values of each marker among the five groups of thyroid cancer considered (normal, adenoma, CPC, FPC and FC tissues).

All calculations were performed using the MedCalc 12.7 software (MedCalc Software, Acacialaan 22, B-8400 Ostend, Belgium) and results were considered statistically significant when *p*-value was ≤0.05. The statistical analysis was carried out considering nuclear and cytoplasmic protein expression.

The Wilcoxon signed-rank test was used to study the correlation between the nuclear and cytoplasmic expression in the *HOX* 13 paralogous group genes because of nonparametric and paired values.

## 5. Conclusions

Cytological evaluation is the first approach to thyroid nodules and most cases are easily categorized as benign or malignant through simple morphology. Differential diagnosis of benign and malignant nodules is sometime however, very difficult, particularly in cytological smears with a clear follicular pattern and patients are subjected to thyroid lobectomy. Thus, the use of *HOX A13* and *HOX B13* expression could enormously help in differential diagnosis of cytologic samples. Its use could also be applied on histological samples, in the definition of well differentiated neoplasias not easily defined as benign or malignant.

Our data may represent an important element for the development of potential therapies targeted against the activities of *HOX* genes. In fact, antisense oligonucleotide (ASO), small interfering RNA (siRNA) [47] and post-translational interaction between HOX proteins and their PBX cofactors, able to block both *in vivo* and *in vitro* tumor cell growth and proliferation, have been recently described [47].

## Supplementary Information



## Figures and Tables

**Table 1:** Clinic pathological features of thyroid cancer patients.

Gender	M	12 (24)
	F	38 (76)

Age	<=45	29 (58)
	46+	21 (42)

HISTOTYPE	CPC	26 (52)
	FPC	17 (34)
	FC	7 (14)

STAGE	I	29 (58)
	II	2 (4)
	III	13 (26)
	IV	6 (12)

**Table 2:** Cytoplasmic *versus* nuclear expression distribution of *HOX A13*, *HOX B13*, *HOX C13* and *HOX D13*. Wilcoxon Signed Ranks Test.

Wilcoxon Signed Ranks Test		

Ranks	N	*p*-Value
*HOX A13*		

Cytoplamic_Expression < Nuclear_Expression	57	<0.001
Cytoplamic_Expression > Nuclear_Expression	1	
Cytoplamic_Expression = Nuclear_Expression	4	

*HOX B13*		

Cytoplasmic_Expression < Nuclear_Expression	3	<0.001
Cytoplasmic_Expression > Nuclear_Expression	47	
Cytoplasmic_Expression = Nuclear_Expression	12	

*HOX C13*		

Cytoplasmic_Expression < Nuclear_Expression	21	0.19
Cytoplasmic_Expression > Nuclear_Expression	29	
Cytoplasmic_Expression = Nuclear_Expression	13	

*HOX D13*		

Cytoplasmic_Expression < Nuclear_Expression	4	<0.001
Cytoplasmic_Expression > Nuclear_Expression	50	
Cytoplasmic_Expression = Nuclear_Expression	9	
