# Supplementary material for: Aberrant Expression of Posterior HOX Genes in Well Differentiated Histotypes of Thyroid Cancers
Source: Int J Mol Sci. 2013 Nov 1;14(11):21727–40. doi: 10.3390/ijms141121727 (PMC3856031; doi:10.3390/ijms141121727)
Supplement: Supplementary file 1 [file ijms-14-21727-s001.pdf]

Supplementary Information

Table S1. Nuclear and cytoplasmic paralogous group 13 *HOX* genes expression in normal, adenoma and tumor phenotypes of thyroid. Kruskal Wallis Test (* significant values).

	Median	Normal	Adenoma	Papillary	Papil/Foll	Follicular	Kruskal Wallis Test	
*HOX A13*_nuclEmembr_PRIM	>	0	0	14	11	6	0.000118	* 1
	<=	6	6	12	6	1		
HOX A13 Citop. PRIM.	>	0	1	5	1	1	0.642506	2
	<=	6	5	21	16	6		
HOX B13 Nucl. PRIM.	>	6	6	9	7	2	0.001318	* 3
	<=	0	0	16	10	5		
HOX B13 Citop. PRIM.	>	3	3	13	8	2	0.810714	4
	<=	3	3	12	9	5		
*HOX C13*_nuclEmembr_PRIM	>	0	3	13	7	3	0.002118	* 5
	<=	6	3	13	10	4		
HOX C13 Citop. PRIM.	>	0	2	14	9	4	0.001367	* 6
	<=	6	4	12	8	3		
*HOX D13*_nuclEmembr_PRIM	>	0	1	10	7	3	0.275741	7
	<=	6	5	16	10	4		
HOX D13 Citop. PRIM.	>	0	1	13	10	6	0.00026	* 8
	<=	6	5	13	7	1		

Table S2. Comparison between the nuclear and cytoplasmic expression of paralogous group 13 *HOX* genes associated in pairs. Wilcoxon Signed anks Test.

	Nuclear expression	Direction	Cytoplasmic Expression	Direction
HOX A13 vs. HOX B13	0.033470126	Up	<0.001	Down
HOX A13 vs. HOX C13	0.192198107	Up	<0.001	Down
HOX A13 vs. HOX D13	<0.001	Up	<0.001	Down
HOX B13 vs. HOX C13	0.183660561	Down	<0.001	Up
HOX B13 vs. HOX D13	<0.001	Up	<0.001	Up
HOX C13 vs. HOX D13	<0.001	Up	<0.001	Up

Table S3. Paralogous group 13 *HOX* genes expression in primary tumors versus lymph node metastases. Wilcoxon Signed Ranks Test.

Ranks nuclear	N	*p*-Value	Ranks cytoplasmic	N	*p*-Value
HOX A13			*HOX A13*		
MTX < Primary	8	0.24	MTX < Primary	1	0.33
MTX > Primary	4		MTX > Primary	4	
MTX = Primary	2		MTX = Primary	9	
HOX B13			*HOX B13*		
MTX < Primary	6	0.92	MTX < Primary	5	0.9
MTX > Primary	6		MTX > Primary	5	
MTX = Primary	2		MTX = Primary	4	
HOX C13			*HOX C13*		
MTX < Primary	6	0.8	MTX < Primary	2	0.65
MTX > Primary	7		MTX > Primary	5	
MTX = Primary	1		MTX = Primary	7	
HOX D13			*HOX D13*		
MTX < Primary	5	0.88	MTX < Primary	9	0.47
MTX > Primary	5		MTX > Primary	5	
MTX = Primary	4		MTX = Primary	0	

© 2013 by the authors; licensee MDPI, Basel, Switzerland. This article is an open access article distributed under the terms and conditions of the Creative Commons Attribution license (http://creativecommons.org/licenses/by/3.0/).
